# Structural analysis of the Toll-like receptor 15 TIR domain

**DOI:** 10.1107/S2052252523002956

**Published:** 2023-04-21

**Authors:** Kyung Yeol Ko, Wan Seok Song, Jeongho Park, Geun-Shik Lee, Sung-il Yoon

**Affiliations:** a Division of Biomedical Convergence, Kangwon National University, Chuncheon, 24341, Republic of Korea; b Institute of Bioscience and Biotechnology, Kangwon National University, Chuncheon, 24341, Republic of Korea; c College of Veterinary Medicine, Kangwon National University, Chuncheon, 24341, Republic of Korea; University of Michigan, USA

**Keywords:** TLR15, Toll-like receptors, TIR domains, dimers, protein structure, molecular recognition, X-ray crystallography

## Abstract

Toll-like receptors (TLRs) activate innate immunity in response to pathogen-associated molecular patterns (PAMPs). The ectodomain of a TLR directly senses a PAMP and the intracellular TIR domain dimerizes to initiate a signaling cascade. The TIR domains of TLR6 and TLR10, which belong to the TLR1 subfamily, have been structurally characterized in a dimer, whereas those of other subfamilies, including TLR15, have not been explored at the structural or molecular level. TLR15 is a TLR unique to birds and reptiles that responds to virulence-associated fungal and bacterial proteases. To reveal how the TLR15 TIR domain (TLR15_TIR_) triggers signaling, the crystal structure of TLR15_TIR_ was determined in a dimeric form and a mutational study was performed. TLR15_TIR_ forms a one-domain structure in which a five-stranded β-sheet is decorated by α-helices, as shown for TLR1 subfamily members. TLR15_TIR_ exhibits substantial structural differences from other TLRs at the BB and DD loops and αC2 helix that are involved in dimerization. As a result, TLR15_TIR_ is likely to form a dimeric structure that is unique in its intersubunit orientation and the contribution of each dimerizing region. Further comparative analysis of TIR structures and sequences provides insights into the recruitment of a signaling adaptor protein by TLR15_TIR_.

## Abstract 2

The intracellular TIR domain of TLR15 is likely to assemble into a unique dimer with distinct structures for the BB and DD loops and αC2 helix.

## 1. Introduction

Toll-like receptors (TLRs) play a key role in the innate immunity of vertebrates (Duan *et al.*, 2022 ▸). TLRs sense the highly conserved molecular patterns of pathogens and recruit signaling adaptor proteins, such as MAL and MyD88, for the expression of proinflammatory cytokines or antiviral interferons, promoting innate immune responses to pathogens (Takeda *et al.*, 2003 ▸; Gay *et al.*, 2014 ▸). Ten TLRs (TLR1–TLR10) have been identified in humans and each of them detects distinct pathogen-associated molecular patterns. For example, TLR3 recognizes viral dsRNA, whereas TLR4 and TLR5 interact with the lipopolysaccharide molecule of Gram-negative bacteria and the flagellin protein of flagellated bacteria, respectively (Yoon *et al.*, 2012 ▸; Song *et al.*, 2017 ▸; Liu *et al.*, 2008 ▸; Park *et al.*, 2009 ▸). Ligand-mediated TLR activation has been extensively studied to develop antimicrobial or anticancer vaccines and therapeutics (Baxevanis *et al.*, 2013 ▸; Connolly & O’Neill, 2012 ▸). Indeed, a TLR4 agonist, mono­phospho­ryl lipid A, has been used as an adjuvant of papilloma­virus vaccine (Giannini *et al.*, 2006 ▸).

A TLR consists of an ectodomain and an intracellular domain (TIR domain, TLR_TIR_ domain), which are linked by a single-pass transmembrane helix (Asami & Shimizu, 2021 ▸). The ectodomain of TLR adopts a horseshoe-shaped leucine-rich repeat structure and directly interacts with pathogen-derived molecules (Botos *et al.*, 2011 ▸). Upon agonist binding, all TLR ectodomains that have been structurally characterized (TLR1–TLR9) assemble into a tail-to-tail dimer, in which their C-terminal regions are located in close proximity (Yoon *et al.*, 2012 ▸; Ohto *et al.*, 2015 ▸; Park *et al.*, 2009 ▸; Liu *et al.*, 2008 ▸; Jin *et al.*, 2007 ▸). This structurally specific dimeric assembly of the TLR ectodomain seems to promote TIR dimerization and subsequent intracellular signaling. The TLR_TIR_ domain forms a five-stranded β-sheet that is surrounded by multiple α-helices (Nyman *et al.*, 2008 ▸; Jang & Park, 2014 ▸; Xu *et al.*, 2000 ▸; Lushpa *et al.*, 2021 ▸). Notably, the TLR_TIR_ domain also dimerizes with a low affinity, given that a dimer was identified at a low level in solution (Jang & Park, 2014 ▸). Thus, the agonist binding-mediated dimerization of the TLR ectodomain is expected to shift the monomer–dimer equilibrium of the TLR_TIR_ domain in favor of a dimeric form. However, dimerization has been addressed only for the TIR domains of TLR6 and TLR10 that belong to the TLR1 subfamily (Nyman *et al.*, 2008 ▸; Jang & Park, 2014 ▸). Thus, it is unclear whether all TLR_TIR_ domains dimerize with an identical organization using similar interfaces.

Vertebrates express a different set of TLRs depending on the taxa. Humans produce TLR1–TLR10, whereas mice express TLR1–TLR9 and TLR11–TLR13. In chickens, TLR1–TLR5 and TLR7 are found. However, chickens lack TLR8 and TLR9 and contain an additional TLR gene that encodes TLR15 (Rehman *et al.*, 2021 ▸). TLR15 is not observed in mammals and is unique to avian and reptilian organisms. In a phylogenetic analysis, bird and reptile TLR15 proteins form a single subfamily that is distinct from the other seven TLR subfamilies (TLR1, TLR3, TLR4, TLR5, TLR7, TLR11 and TLR13 subfamilies) even though the TLR15 subfamily is evolutionarily close to the TLR1 subfamily (Liu *et al.*, 2020 ▸). TLR15 has been reported to be a unique receptor that senses virulence-associated fungal and bacterial proteases (Zoete *et al.*, 2011 ▸). The microbial protease proteolytically cleaves TLR15, inducing TLR15-mediated signaling. Despite the biological significance of TLR15 in defense against pathogens, TLR15 has not been characterized structurally, and the molecular mechanism for TLR15 signaling has never been experimentally addressed. Here, we present the dimeric structure of the TLR15 TIR (TLR15_TIR_) domain, which would represent an activated TLR_TIR_ organization.

## 2. Materials and methods

### 2.1. Construction of the protein-expression plasmid

To construct a TLR15_TIR_ protein-expression plasmid, the DNA region that encodes TLR15_TIR_ protein (residues 699–868) was amplified by polymerase chain reaction (PCR) from the cDNA library of *Gallus gallus* spleen. The PCR product was treated with the BamHI and SalI restriction enzymes, and the resulting DNA fragment was inserted using T4 DNA ligase into a pET49b plasmid that had been modulated to express recombinant protein with a hexahistidine (His_6_) affinity tag and a thrombin digestion site at the N-terminus (Park *et al.*, 2021 ▸). The ligation product was transformed into *Escherichia coli* DH5α cells in the presence of kanamycin. The nucleotide sequence of the insert in the protein-expression plasmid from the transformant was verified by restriction-enzyme digestion and DNA sequencing. TLR15_TIR_ mutagenesis was performed using ssDNA oligomers containing mutation sequences based on the QuikChange site-directed mutagenesis protocol (Agilent).

### 2.2. Protein expression and purification

To obtain the recombinant TLR15_TIR_ protein, the TLR15_TIR_ expression vector was transformed into the *E. coli* strain Rosetta-gami 2 (DE3). The *E. coli* cells containing the TLR15_TIR_ expression plasmid were cultured at 37°C in LB medium. When the optical density of the culture at 600 nm reached 0.6, the culture was supplemented with 1 m*M* iso­propyl β-d-1-thiogalactopyran­oside for TLR15_TIR_ protein overexpression. The cells were further cultured at 18°C for 18 h and harvested by centrifugation. The cell pellet was resuspended in a solution containing 50 m*M* Tris, pH 8.0, 200 m*M* NaCl and 5 m*M* β-mercapto­ethanol, and lysed by sonication. The cell lysate was cleared by centrifugation and incubated with Ni–NTA resin (Qiagen) in the presence of 10 m*M* imidazole at 4°C for 1.5 h. The resin was harvested into an Econo-column (Bio-Rad) and washed with a solution containing 50 m*M* Tris, pH 8.0, 200 m*M* NaCl, 5 m*M* β-mercapto­ethanol, and 10 m*M* imidazole. The TLR15_TIR_ protein was eluted using a solution containing 50 m*M* Tris, pH 8.0, 200 m*M* NaCl, 5 m*M* β-mercapto­ethanol and 250 m*M* imidazole. The eluted TLR15_TIR_ protein was dialyzed against a solution containing 20 m*M* HEPES, pH 7.4, 150 m*M* NaCl and 5 m*M* β-mercapto­ethanol. The resulting His_6_-tagged TLR15_TIR_ protein was treated with thrombin to remove the His_6_ affinity tag. The tag-free TLR15_TIR_ protein was purified in the presence of β-mercapto­ethanol by anion-exchange chromatography using a Mono Q 10/100 column (GE Healthcare) for crystallization or by gel-filtration chromatography using a Superdex 200 16/600 column (GE Healthcare) for dimerization analysis. TLR15_TIR_ protein was also obtained in the absence of β-mercapto­ethanol from cell lysis through the final purification step of gel-filtration chromatography and used to generate the gluta­thione-adduct crystal structure of TLR15_TIR_.

### 2.3. Crystallization and X-ray diffraction

The TLR15_TIR_ protein was crystallized using a sitting-drop vapor-diffusion method by equilibrating a 1 µl drop of protein and crystallization reagent over a well solution at 18°C. Initial crystals were obtained using MCSG crystallization screens, and the crystallization conditions were optimized. The TLR15_TIR_ protein that was obtained in the presence of β-mercapto­ethanol was crystallized in 2.0 *M* ammonium sulfate, 0.1 *M* NaCl and 0.1 *M* sodium cacodylate, pH 6.5. The TLR15_TIR_ protein that was purified in the absence of β-mercapto­ethanol was crystallized in 1.2 *M* dipotassium hydrogen phosphate, 0.65 *M* sodium di­hydrogen phosphate and 0.1 *M* sodium acetate, pH 4.5. The resulting TLR15_TIR_ crystal was cryoprotected using glycerol and flash-cooled under a gaseous nitro­gen stream at −173°C. The X-ray diffraction of the crystal was performed at the Pohang Accelerator Laboratory, beamline 7A. The diffraction data were processed using the *HKL*2000 program (Otwinowski & Minor, 1997 ▸).

### 2.4. Structure determination

The crystal structure of TLR15_TIR_ was determined by molecular replacement with the *Phaser* program using the structure of the TLR1 TIR domain (PDB ID 7nuw) as a search model (Lushpa *et al.*, 2021 ▸; McCoy *et al.*, 2007 ▸). The final structure of TLR15 was obtained through iterative cycles of manual building and refinement using the *Coot* and *phenix.refine* programs, respectively (Emsley & Cowtan, 2004 ▸; Adams *et al.*, 2010 ▸).

### 2.5. Gel-filtration chromatography

The oligomeric state of the TLR15_TIR_ protein was analyzed by gel-filtration chromatography using a Superdex 200 10/300 column (GE Healthcare). The column was equilibrated with a running solution containing 20 m*M* HEPES, pH 7.4, 150 m*M* NaCl and 5 m*M* β-mercapto­ethanol, and then loaded with 30 µg of TLR15_TIR_ protein (250 µl) that was purified in the presence of β-mercapto­ethanol. Protein elution was performed using the running solution and monitored by measuring the absorbance at 280 nm. The fractions obtained from gel-filtration chromatography were analyzed by SDS–PAGE and silver staining.

## 3. Results and discussion

### 3.1. Overall structure of the TLR15_TIR_ domain

**Figure 1:**
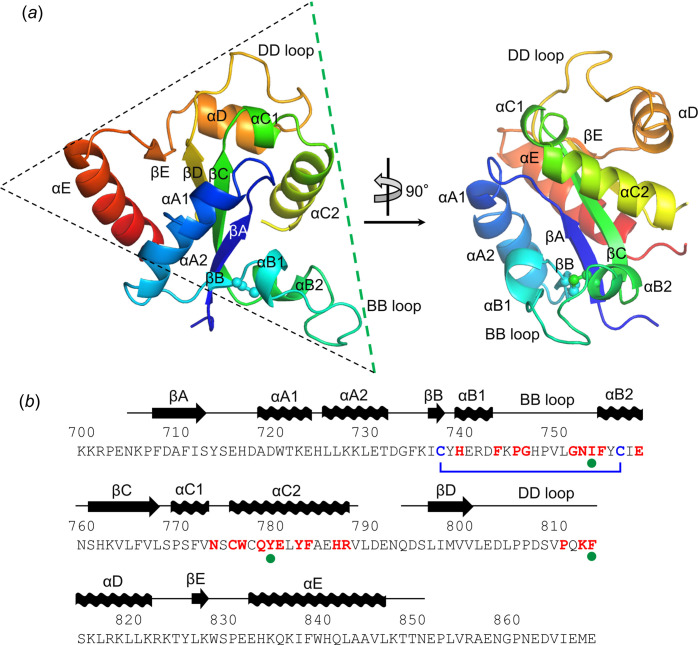
Monomer structure and sequence of the TLR15_TIR_ domain. (*a*) Overall structure of a TLR15_TIR_ monomer. The TLR15_TIR_ monomer structure is shown as rainbow ribbons (N-terminus, blue; C-terminus, red). The triangular shape of the TLR15_TIR_ structure is outlined by a dashed triangle, and the side of the TLR15_TIR_ triangle that participates in dimerization is colored green. The Cys737 and Cys756 residues that form an intramolecular di­sulfide bond are represented by ball-and-stick models. (*b*) The TLR15_TIR_ sequence. The secondary structures of TLR15_TIR_ are represented by arrows and waves (β-strands and α-helices, respectively) above the TLR15_TIR_ sequence. The dimerization interface residues of TLR15_TIR_ are colored red. The Cys737 and Cys756 residues that participate in an intramolecular di­sulfide bond are colored blue and linked by a blue line. The Ile753, Tyr779 and Phe813 residues of TLR15 that were mutated to confirm the TLR15_TIR_ dimerization interface are indicated by green dots below the TLR15_TIR_ sequence.

To provide the structural mechanism whereby TLR15 mediates signaling via its intracellular TIR domain, chicken TLR15_TIR_ (residues 699–868) was expressed using the *E. coli* expression system and purified by chromatographic methods in the presence of β-mercapto­ethanol (see Fig. S1 of the supporting information). The TLR15_TIR_ protein was crystallized using ammonium sulfate at pH 6.5. The TLR15_TIR_ crystals diffracted X-rays up to 1.90 Å resolution. The crystal structure of TLR15_TIR_ was determined by molecular replacement and refined with an *R*_free_ value of 23.3% (Table 1 ▸). The asymmetric unit of the TLR15_TIR_ crystal contains one polypeptide chain of TLR15_TIR_ [Fig. 1 ▸(*a*)]. The TLR15_TIR_ structure covers TLR15 residues 704–788 and 793–850 (Fig. 1 ▸).

The TLR15_TIR_ structure forms a one-domain αβ structure, in which a five-stranded β-sheet (βB–βA–βC–βD–βE) is located in the center of the structure and both faces of the β-sheet are decorated by α-helices (αA1, αA2, αB1, αB2 and αE helices on one face of the β-sheet; αC1, αC2 and αD helices on the other face) [Fig. 1 ▸(*a*)]. Each β-strand is C-terminally appended to one or two α-helices (Fig. 1 ▸). TLR15_TIR_ adopts a triangular shape, whose vertices are occupied by a loop connecting the αB1 and αB2 helices (BB loop), by a loop connecting the βD strand and αD helix (DD loop), or by the αE helix [Fig. 1 ▸(*a*)]. TLR15_TIR_ contains four cysteine residues, two of which (Cys737 and Cys756) form an intramolecular di­sulfide bond that tethers and stabilizes the βB strand and αB2 helix (Fig. 1 ▸).

**Figure 2:**
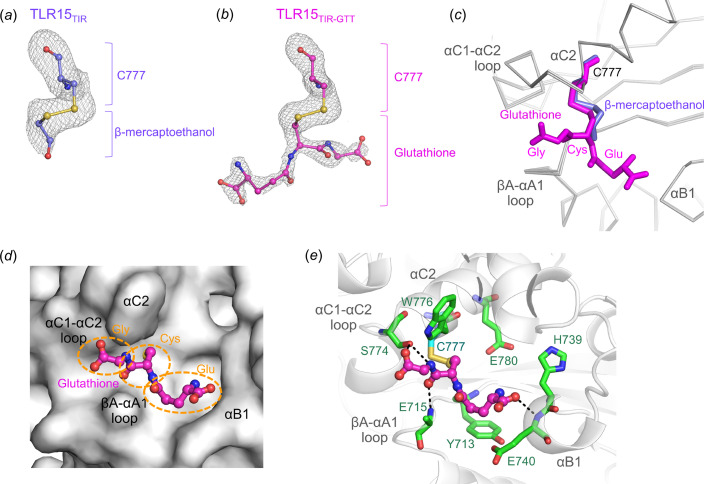
β-Mercapto­ethanol- or gluta­thione-adduct Cys777 residue of TLR15_TIR_. (*a*) An electron-density map (gray meshes; 3.0σ in *F*_o_ − *F*_c_ OMIT map) for the β-mercapto­ethanol-adduct Cys777 residue (carbon, light blue; oxygen, red; nitro­gen, blue; sulfur, yellow) in the TLR15_TIR_ structure. (*b*) An electron-density map (gray meshes; 3.0σ in *F*_o_ − *F*_c_ OMIT map) for the gluta­thione-adduct Cys777 residue (carbon, magenta; oxygen, red; nitro­gen, blue; sulfur, yellow) in the TLR15_TIR-GTT_ structure. (*c*) β-Mercapto­ethanol- and gluta­thione-adduct Cys777 residues (light blue and magenta ball-and-stick models, respectively) from the overlaid TLR15_TIR_ and TLR15_TIR-GTT_ structures (gray Cα traces), respectively. (*d*) Gluta­thione adduct (magenta ball-and-stick model) in the dent of the TLR15_TIR-GTT_ structure (gray surfaces). Gluta­thione consists of Gly, Cys and Glu moieties. (*e*) Interactions between TLR15 and gluta­thione in the TLR15_TIR-GTT_ structure. The gluta­thione molecule that is linked to TLR15 Cys777 (cyan sticks) is shown as a magenta ball-and-stick model. The gluta­thione-binding residues of TLR15 are depicted as green sticks on gray transparent ribbons representing the TLR15_TIR-GTT_ structure. Hydrogen bonds between gluta­thione and TLR15 residues are represented by black dotted lines.

Although the Cys777 residue of TLR15 is not involved in a di­sulfide bond, it exhibited strong electron density over its sulfur atom in the crystal structure of TLR15_TIR_ [Fig. 2 ▸(*a*)]. Given that β-mercapto­ethanol was used for the purification of TLR15_TIR_ and resembles the curved linear shape of the extra electron density, the Cys777 residue was modeled as an adduct with β-mercapto­ethanol. To obtain a TLR15_TIR_ structure that was not modified by β-mercapto­ethanol, TLR15_TIR_ protein was purified under nonreducing conditions in the absence of β-mercapto­ethanol, and crystallized using dipotassium hydrogen phosphate and sodium di­hydrogen phosphate at pH 4.5. The new crystal structure, named TLR15_TIR-GTT_, was determined at 1.8 Å resolution with an *R*_free_ value of 24.2%. The TLR15_TIR-GTT_ structure is essentially identical to the β-mercapto­ethanol-adduct TLR15_TIR_ structure with a root-mean-square deviation (RMSD) value of 0.22 Å (Fig. S2). Surprisingly, in the TLR15_TIR-GTT_ structure, the Cys777 residue also exhibited extra electron density next to its sulfur atom [Fig. 2 ▸(*b*)]. However, the extra electron density does not resemble the short linear β-mercapto­ethanol molecule and instead adopts a three-armed starfish-like shape, which resembles that of gluta­thione, another thiol-containing chemical. Therefore, the Cys777 residue was built as a gluta­thione adduct in the TLR15_TIR-GTT_ structure. The central part of gluta­thione that corresponds to a Cys moiety adopts a similar conformation to that of β-mercapto­ethanol from the β-mercapto­ethanol-adduct TLR15_TIR_ structure [Fig. 2 ▸(*c*)]. Because gluta­thione is the most prevalent low-molecular-weight thiol in the cytosol of cells, including *E. coli*, at millimolar concentrations, the TLR15_TIR_ Cys777 residue seems to have captured gluta­thione during recombinant expression in *E. coli* cells (Forman *et al.*, 2009 ▸). For the same reason, the TLR15 Cys777 residue is expected to form an adduct with gluta­thione even when TLR15 is naturally expressed in chicken cells. Because the TLR15 Cys777 residue is readily modified by β-mercapto­ethanol or gluta­thione, we propose that the Cys777 residue is highly reactive. Consistently, the gluta­thione adduct is inserted into the dent of TLR15_TIR_ that is surrounded by residues from βA—αA1 loop, αB1 helix, αC1—αC2 loop and αC2 helix [Fig. 2 ▸(*d*)]. The Gly and Cys moieties of gluta­thione are located between the αC2 helix and βA—αA1 loop, and make contact with the Glu715, Ser774 and Trp776 residues, forming hydrogen bonds with the Glu715 and Ser774 residues [Fig. 2 ▸(*e*)]. The Glu moiety of gluta­thione is directed toward the αB1 helix and interacts with the Tyr713 and Glu715 residues from the βA—αA1 loop as well as the His739 and Glu740 residues from the αB1 helix, forming hydrogen bonds with the Tyr713 and Glu740 residues. These gluta­thione-binding residues are absolutely conserved in TLR15 orthologs (Fig. S3).

Interestingly, in the crystal structure of the human MAL TIR domain, the Cys177 residue of MAL that corresponds to the TLR15 Cys777 residue forms an adduct with di­thio­threitol containing two thiol groups and is linked to the neighboring cysteine residue (Cys91) through this di­thio­threitol molecule (Valkov *et al.*, 2011 ▸; Lin *et al.*, 2012 ▸). However, TLR15 contains a serine residue (Ser712) in place of the MAL Cys91 residue and thus does not seem to prefer di­thio­threitol as a Cys777 modifier.

### 3.2. Dimeric structure of TLR15_TIR_

**Figure 3:**
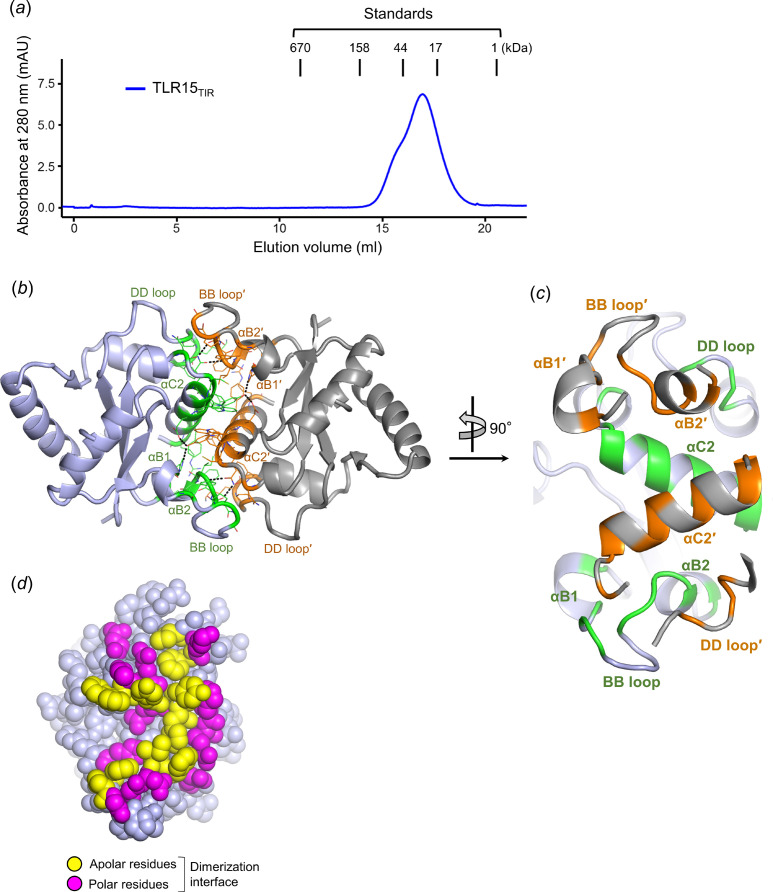
TLR15_TIR_ dimerization. (*a*) Gel-filtration chromatography analysis of the TLR15_TIR_ protein. (*b*) The dimeric structure of TLR15_TIR_. Two TLR15_TIR_ chains (TLR15_TIR_ and its binding partner TLR15_TIR_′) that form a dimer are depicted as light blue and gray ribbons. The dimerization interface residues of TLR15_TIR_ and TLR15_TIR_′ are colored green and orange, respectively, in the ribbon diagram, and also shown as green and orange lines, respectively. Intersubunit hydrogen bonds are represented by black dotted lines. (*c*) The dimerization interface of TLR15_TIR_. The TLR15_TIR_ and TLR15_TIR_′ chains are shown as light blue and gray ribbons, respectively. The dimerization interface residues of TLR15_TIR_ and TLR15_TIR_′ are colored green and orange, respectively. A detailed view of the dimerization interface is shown in Fig. S4. (*d*) Apolar and polar TLR15_TIR_ residues (yellow and magenta spheres, respectively) in the dimerization interface.

The TIR domains of TLR1 subfamily members (TLR6 and TLR10) have been shown to form a dimer with a low dimerization affinity (Nyman *et al.*, 2008 ▸; Jang & Park, 2014 ▸). To address the oligomeric state of the TLR15_TIR_ domain, the TLR15_TIR_ protein was analyzed by gel-filtration chromatography. TLR15_TIR_ generated a major peak, corresponding to a TLR15_TIR_ monomer, with a dimer shoulder, suggesting that TLR15_TIR_ exists dominantly as a monomer and is able to form a low level of dimer [Fig. 3 ▸(*a*)].

To provide insights into the dimeric architecture of TLR_TIR_, TLR15_TIR_ dimerization was inspected in the crystal. Three dimers (>100 Å^2^ buried surface area) are created via crystallographic rotational or translational symmetries. Among the three dimers, only one dimer exhibits a twofold rotational symmetry, with a significant buried surface area (∼1110 Å^2^ on each monomer) [Fig. 3 ▸(*b*)]. In the twofold dimer, the dimerization interface is primarily located on one side of the triangular TLR15_TIR_ structure that is mainly lined with the BB loop, αC2 helix and DD loop. The dimerization interface consists of 22 residues from three regions of TLR15_TIR_ (the BB region including the BB loop and its flanking αB1 and αB2 helices; the αC2 region including the αC2 helix and its N-terminal loop, αC1—αC2 loop; and the DD region corresponding to the DD loop) [Figs. 3 ▸(*c*) and S4]. TLR15_TIR_ dimerization is mediated by diverse types of interactions, such as hydrogen bonds, hydro­phobic interactions and van der Waals interactions. Apolar residues are primarily located in the center of the dimerization interface, mediating hydro­phobic interactions [Fig. 3 ▸(*d*)]. Polar residues are mainly positioned in the periphery, enclosing the hydro­phobic central interface. The dimerization interface residues of chicken TLR15_TIR_ are highly conserved in orthologs from birds and reptiles (Fig. S3).

The αC2 region makes the greatest contributions to dimerization with a buried surface area of ∼650 Å^2^ from ten residues [Figs. 3 ▸(*b*), 3 ▸(*c*) and S4]. The αC2 helix is located in the middle of the dimerization interface and makes symmetrical contacts with its counterpart helix (αC2′ helix; the prime denotes the dimerization partner) from the dimerization chain in a diagonal manner, mediating major dimerizing interactions. In addition to this primary interhelix interaction, the αC2 region recognizes the BB′ region using its N-terminal residues. The BB′ region also interacts with the DD loop in addition to the αC2 region. The BB and DD regions are involved in dimerization at lower levels (nine residues with a buried surface area of ∼340 Å^2^ and three residues with a buried surface area of ∼130 Å^2^, respectively) than the αC2 region (ten residues with a buried surface area of ∼650 Å^2^).

**Figure 4:**
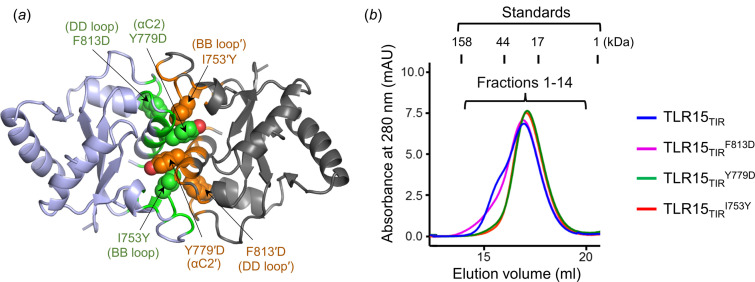
Mutational analysis to confirm the dimerization interface of TLR15_TIR_. (*a*) Dimerization interface residues of TLR15_TIR_ mutated to confirm that the BB loop, αC2 helix and DD loop are involved in TLR15_TIR_ dimerization. The mutated residues (Ile753, Tyr779 and Phe813) are shown as green or orange spheres in the TLR15_TIR_ dimer structure (light blue or gray ribbons) and are labeled with mutations (I753Y, Y779D and F813D). (*b*) Gel-filtration chromatography analysis of the dimerization-deficient TLR15_TIR_ mutants in comparison with TLR15_TIR_. The SDS–PAGE analysis of the gel-filtration chromatography fractions 1–14 is shown in Fig. S5.

To verify the dimerization interface of TLR15_TIR_, we mutated the TLR15 Tyr779 residue to aspartate. Tyr779 is located in the middle of the αC2 helix in the center of the dimerization interface [Figs. 4 ▸(*a*) and S4]. The side chain of the TLR15 Tyr779 residue is inserted into a small cavity that is created by the His739′, Ile753′, Glu780′ and Phe783′ residues. In the cavity, the Tyr779 residue makes hydro­phobic interactions with Ile753′ and Phe783′ and forms hydrogen bonds with His739′ and Glu780′. To disrupt these Tyr779-mediated interactions, the Tyr779 residue was replaced with aspartate. The negative charge at Y779D is expected to abolish the hydro­phobic interactions and mediate electrostatic repulsion from the negatively charged Glu780′ residue. Indeed, in gel-filtration chromatography, the Y779D mutant generated only a monomer peak, without the left shoulder corresponding to the dimer [Figs. 4 ▸(*b*) and S5]. Moreover, when the Ile753 residue from the BB loop was mutated to a larger residue, tyrosine, to reduce shape complementarity, dimerization was not observed, indicating the critical role of the BB loop in dimerization. Furthermore, the Phe813 residue from the DD loop that participates in hydro­phobic interactions with Ile753′ and Phe754′ was also replaced with aspartate. The F813D mutation partially disrupted dimer formation, suggesting that the DD loop is also involved in dimerization. Collectively, our structural and mutational analyses suggest that TLR15_TIR_ employs the BB, αC2 and DD regions for dimerization.

### 3.3. Comparative analysis of TLR_TIR_ domains

The TLR_TIR_ domain has been structurally defined for TLR1, TLR2, TLR6 and TLR10, all of which belong to the TLR1 subfamily. TLR15 is closely related to these TLR1 subfamily members with high sequence identities (40–46%) and a similar secondary structure topology (Figs. S1 and S6). However, the TLR15_TIR_ structure exhibits relatively high RMSD values (1.69–1.81 Å for ∼125 Cα atoms) with the structures of the TLR1, TLR2, TLR6 and TLR10 TIR domains (TLR1_TIR_, TLR2_TIR_, TLR6_TIR_ and TLR10_TIR_, respectively), primarily because of large structural deviations at the BB loop, αB2 helix, αC2 helix and DD loop (Fig. S6).

**Figure 5:**
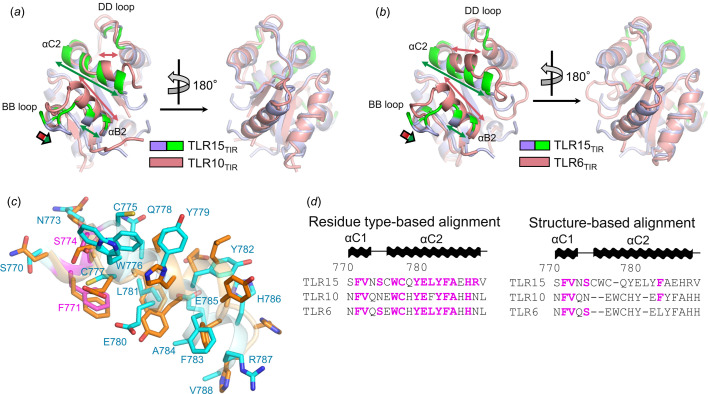
Structural comparison of TLR15_TIR_ with TLR10_TIR_ and TLR6_TIR_. (*a*) Overlaid monomer structures of TLR15_TIR_ (dimerization interface, green; other regions, light blue) and TLR10_TIR_ (salmon; PDB ID 2j67; Nyman *et al.*, 2008 ▸). The lengths of the αB2 and αC2 helices are indicated by double-headed arrows (TLR15_TIR_, green; TLR10_TIR_, salmon). Structural differences at the BB loop are indicated by a thick single-headed arrow. (*b*) Overlaid monomer structures of TLR15_TIR_ (dimerization interface, green; other regions, light blue) and TLR6_TIR_ (salmon; PDB ID 4om7; Jang & Park, 2014 ▸). The lengths of the αB2 and αC2 helices are indicated by double-headed arrows (TLR15_TIR_, green; TLR6_TIR_, salmon). The structural differences at the BB loop are indicated by a thick single-headed arrow. (*c*) TLR15_TIR_ and TLR6_TIR_ (PDB ID 4om7) structures at the αC2 helix and its neighboring regions. The TLR15 residues that are identical to the TLR6 residues are shown as magenta sticks, and those that differ are depicted as cyan sticks. TLR6 residues are represented by orange sticks. (*d*) Residue type-based and structure-based alignments of TLR15, TLR10 and TLR6 sequences at the αC2 helix and its neighboring regions. The TLR10 and TLR6 residues that are identical to those of TLR15 are colored magenta.

The most pronounced structural differences between TLR15 and TLR1 subfamily members are observed at the αC2 helix [Figs. 5 ▸(*a*) and 5 ▸(*b*)]. The TLR15 αC2 helix (13 residues) is substantially longer than those of the TLR1 subfamily members (4–7 residues) (Fig. S1). The straight extension of the TLR15_TIR_ αC2 helix allows its N-terminal residues (Cys775 and Trp776) to form a protrusion that is not found in TLR1 subfamily members [Fig. 5 ▸(*c*)]. Therefore, in structural overlays, the TLR15_TIR_ Cys775 and Trp776 residues lack structurally equivalent residues in TLR1 subfamily members, although the TLR15_TIR_ Trp776 residue is aligned with the invariant tryptophan residue of TLR1 subfamily members at the αC2 helix in residue type-based sequence alignment [Fig. 5 ▸(*d*)]. Similar discrepancies between structure-based alignment and residue type-based sequence alignment are observed throughout the αC2 helix. Although the αC2 helix residues of TLR15 are well aligned to the sequences of TLR1 subfamily members with identical residue types without addition and deletion in residue type-based sequence alignment, most TLR15 αC2 helix residues are structurally overlaid on different residue types from TLR1 subfamily members [Figs. 5 ▸(*c*) and 5 ▸(*d*)]. For example, the TLR15 Tyr779 residue is structurally equivalent to histidine or lysine rather than to the next tyrosine residue in TLR1 subfamily members. Consistent with this structural finding, both the TLR15 Tyr779 residue and its structurally equivalent histidine or lysine residues of TLR1 subfamily members are exposed to solvent in monomer structures and are involved in dimerization in dimer structures, whereas the next tyrosine residue of TLR1 subfamily members is buried in the hydro­phobic core and does not contribute to dimerization. Another structural distinction of the TLR15_TIR_ αC2 helix is its closer positioning to the main body of the TLR15_TIR_ structure with a different orientation. Therefore, the TLR15 residues at the αC2 helix adopt conformations and locations distinct from those of TLR1 subfamily members, contributing to the unique dimerization of TLR15_TIR_. Interestingly, the αC2 helix of TLR15_TIR_ resembles those of signaling adaptor molecules, such as MAL and MyD88, in local structures more closely than those of TLR1 subfamily members, although TLR15_TIR_ exhibits larger deviations from the TIR domains of MAL and MyD88 in the overall structures (RMSD, 2.3–2.4 Å) and sequences (sequence identities, 18–27%) (Lin *et al.*, 2012 ▸; Clabbers *et al.*, 2021 ▸).

Because the αC2 helix makes contact with the αB2 helix and DD loop, the structural differences between TLR15 and TLR1 subfamily members at the αC2 helix are accompanied by those of the αB2 helix, its neighboring BB loop and the DD loop. In addition, the TLR15_TIR_ structure has a shorter αB2 helix than those of TLR1 subfamily members, and the BB loop that is located before the αB2 helix adopts a more extended loop structure that is inclined toward the αB2 helix. Interestingly, the BB and DD loops and αB2 and αC2 helices of TLR15_TIR_, which exhibit large structural differences from those of TLR1 subfamily members, are involved in TLR15_TIR_ dimerization.

### 3.4. Comparative analysis of TLR_TIR_ dimerization

**Figure 6:**
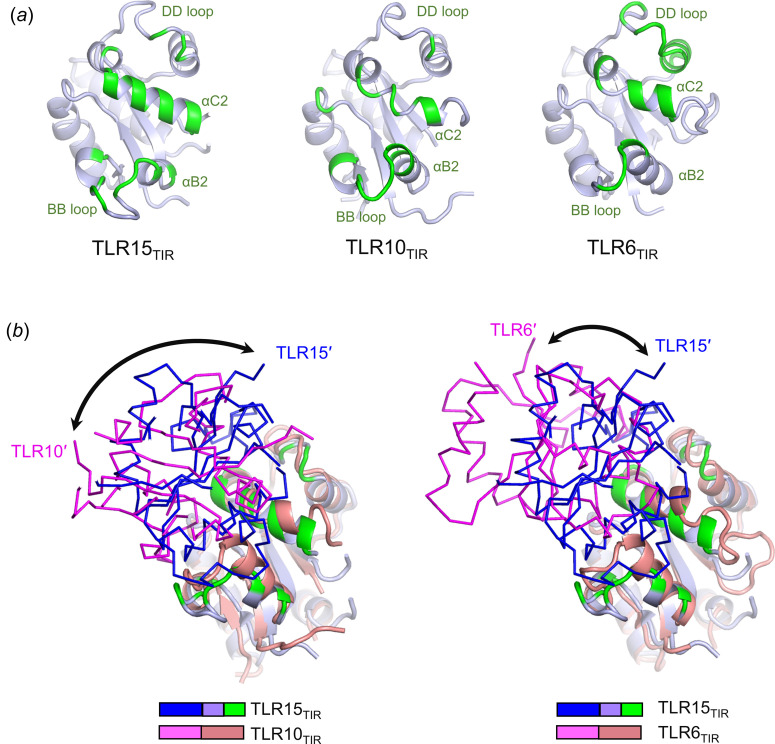
Comparison of the TLR15_TIR_, TLR10_TIR_ and TLR6_TIR_ structures in the dimerization interfaces and intersubunit organizations. (*a*) Dimerization interfaces of TLR15_TIR_, TLR10_TIR_ (PDB ID 2j67) and TLR6_TIR_ (PDB ID 4om7). Dimerization interface residues are colored green in the TLR_TIR_ structure (light blue ribbons). (*b*) Dimeric organization of TLR15_TIR_ (light blue ribbons and blue Cα traces) in comparison with that of TLR10_TIR_ (left; salmon ribbons and magenta Cα traces; PDB ID 2j67) or TLR6_TIR_ (right; salmon ribbons and magenta Cα traces; PDB ID 4om7). Dimerization interface residues are highlighted in green in the TLR15_TIR_ monomer structure shown as ribbons. The TLR_TIR_ dimer structures are superimposed using the TLR_TIR_ monomers shown as ribbons. Orientation differences are indicated by double-headed arrows.

The TLR_TIR_ dimerization of TLR6 and TLR10 has been addressed by previous structural studies (Nyman *et al.*, 2008 ▸; Jang & Park, 2014 ▸). Both TLR6_TIR_ and TLR10_TIR_ form a homodimer using the BB, αC2 and DD regions, as observed for TLR15_TIR_ [Fig. 6 ▸(*a*)]. However, because of structural differences in the BB, αC2 and DD regions, the TLR_TIR_ domains dimerize in distinct intersubunit orientations with the different contributions of each region (Fig. 6 ▸).

TLR15_TIR_ dimerization is primarily mediated by the αC2 region (59%), with the second contribution by the BB region (30%) [Fig. 6 ▸(*a*)]. In contrast, in the TLR10_TIR_ dimer, the BB region makes the largest contribution to dimerization, with 62% of the dimerization interface, and the αC2 region is responsible for 32%, with a negligible contribution from the DD region. TLR6_TIR_ dimerizes mainly using the αC2 region (59%), as observed for the TLR15_TIR_ dimer, but with the second highest contribution from the DD region (25%), unlike TLR15 and TLR10. Moreover, the involvement of each residue in dimerization differs between TLRs. For example, the TLR15 Ile753 residue from the BB loop and its structurally equivalent residue in TLR10 (Ile682) are involved in dimerization, whereas their equivalent residue in TLR6 (Ile688) is not located in the dimerization interface. Furthermore, the TLR15 Phe813 residue from the DD loop contributes to dimerization, similar to TLR6 Tyr748, whereas the equivalent residue in TLR10 (Tyr742) does not. As a result of the unique dimerization contribution and conformation of each dimerization region, TLR15_TIR_ differs in intersubunit orientation from TLR10_TIR_ and TLR6_TIR_ [Fig. 6 ▸(*b*)].

### 3.5. Implication of the TLR15_TIR_ structure in MyD88 recruitment

TLRs selectively recruit intracellular adaptor proteins, including MAL, MyD88, TRAM and TRIF, upon dimerization for intracellular signaling (Gay *et al.*, 2014 ▸). For example, a TLR4 TIR dimer forms a large complex, called the TIR signalosome, with multiple copies of the MAL and MyD88 TIR domains, allowing MyD88 to cluster with IRAK4 and IRAK2 using its death domain (Ve *et al.*, 2017 ▸; Clabbers *et al.*, 2021 ▸; Lin *et al.*, 2010 ▸). Although it is unclear which adaptor protein TLR15 binds, RNA interference analysis has suggested that TLR15 signaling depends on MyD88 (Ciraci & Lamont, 2011 ▸). The BB loop of TLR TIR has been suggested to play a key role in MyD88 recruitment (Xu *et al.*, 2000 ▸). In particular, a proline residue at the BB loop is invariant in MyD88-dependent TLRs and was shown to be required for TLR signaling (Hasan *et al.*, 2005 ▸). TLR3, which recruits TRIF instead of MyD88, contains alanine in place of proline, and mutation of the alanine residue to proline switches the signaling adaptor specificity of TLR3 from TRIF to MyD88 (Verstak *et al.*, 2013 ▸). TLR15 also contains a proline residue (Pro745) at the BB loop [Fig. 1 ▸(*b*)]. Pro745 is located at the edge of the dimerization interface and is exposed to solvent in the dimer structure, presumably to recruit an adaptor protein (Fig. S4). These observations allow us to propose that TLR15 signals through MyD88.

**Figure 7:**
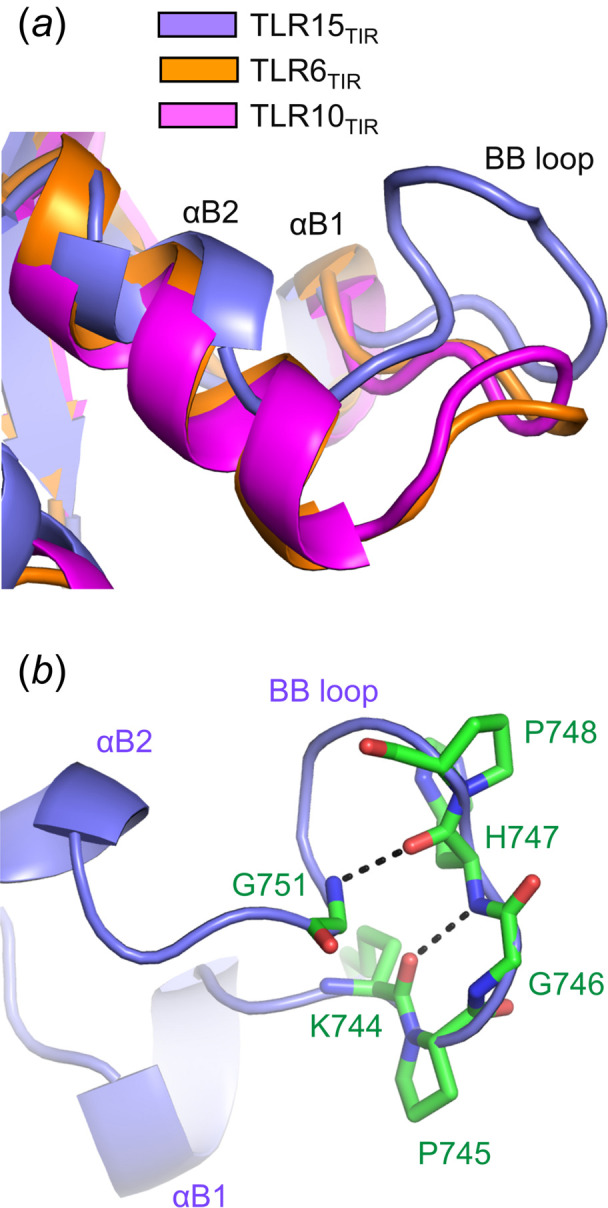
Extended protruding structure of the TLR15_TIR_ BB loop. (*a*) The BB loop and its flanking αB1 and αB2 helices in TLR15_TIR_ (light blue ribbons), TLR6_TIR_ (orange ribbons; PDB ID 4om7) and TLR10_TIR_ (magenta ribbons; PDB ID 2j67). (*b*) Two proline residues at the TLR15_TIR_ BB loop. The two proline residues and their neighboring residues at the TLR15_TIR_ BB loop are shown as green sticks on the ribbon diagram of the TLR15_TIR_ structure, and main-chain hydrogen bonds at the BB loop are represented by dashed lines.

The BB loop of TLR15_TIR_ displays a unique structure that is not found in other TLRs. The TLR15_TIR_ BB loop adopts a more extended structure because the αB2 helix that precedes the BB loop is shorter [Fig. 7 ▸(*a*)]. Moreover, this extended BB loop of TLR15_TIR_ forms a distinct protrusion (residues 744–751) that is stabilized by two proline residues (Pro745 and Pro748) [Figs. 7 ▸(*b*) and S1]. The first proline residue (TLR15 Pro745) is conserved in TLR15 and TLR1 subfamily members, and mediates a β-turn along with its following invariant glycine residue (TLR15 Gly746). The second proline residue (TLR15 Pro748) is found only in TLR15, and induces a turn through a hydrogen bond between His747 and Gly751. As a result, the central region of the TLR15_TIR_ BB loop that exhibits a high sequence deviation from that of TLR1 subfamily members is exposed to solvent in the dimer structure, presumably to bind a signaling adaptor protein. The unique structure of the TLR15_TIR_ BB loop suggests that TLR15 interacts with an adaptor protein via distinct binding affinity or orientation.

In conclusion, we have presented the highly potential dimeric architecture of TLR15_TIR_ based on structural and mutational studies on TLR15_TIR_. Moreover, our comparative analysis of diverse TLR_TIR_ structures suggests that TLR1 and TLR15 subfamilies use similar regions for dimerization but with different contributions. Further structural studies on TIR domains from other TLR subfamilies are required to determine whether our conclusion extends to all TLR_TIR_ domains. Furthermore, our comparative analysis provides insights into the TLR15-mediated recruitment of a signaling adaptor protein.

## Supplementary Material

Supporting figures. DOI: 10.1107/S2052252523002956/jt5065sup1.pdf

PDB reference: Toll-like receptor 15 TIR domain (gluta­thione adduct), 7ylg

PDB reference: Toll-like receptor 15 TIR domain (β-mercapto­ethanol adduct), 7ylf

## Figures and Tables

**Table 1:** Crystallographic statistics of the TLR15_TIR_ structures

	TLR15_TIR_	TLR15_TIR-GTT_
Data collection		
Space group	*P*3_2_21	*P*3_2_21
Cell parameters (Å)	*a* = *b* = 64.30, *c* = 95.78	*a* = *b* = 64.63, *c* = 94.91
Wavelength (Å)	0.9793	0.9793
Resolution (Å)	30.00–1.90	30.00–1.80
Highest resolution (Å)	1.93–1.90	1.83–1.80
No. unique reflections	18382 (902)	21418 (1056)
*R*_merge_	0.068 (0.674)	0.073 (1.424)
*R*_meas_	0.074 (0.741)	0.079 (1.534)
*R*_p.i.m._	0.029 (0.293)	0.029 (0.558)
CC_1/2_	0.996 (0.778)	0.996 (0.822)
〈*I*/σ(*I*)〉	32.9 (2.3)	39.8 (2.3)
Completeness (%)	98.7 (98.8)	97.8 (99.5)
Redundancy	5.4 (5.5)	7.2 (7.2)
		
Refinement		
Resolution (Å)	30.00–1.90	30.00–1.80
No. reflections (work)	17499	20236
No. reflections (test)	838	1085
*R*_work_ (%)	20.1	21.4
*R*_free_ (%)	23.3	24.2
No. atoms	1240	1225
Average *B* value (Å^2^)	41.5	40.4
RMSD bonds (Å)	0.007	0.009
RMSD angles (°)	0.82	0.87
Ramachandran favored (%)	99.3	100.0
Ramachandran outliers (%)	0.0	0.0
PDB ID	7ylf	7ylg
