# Supplementary material for: Structural analysis of the Toll-like receptor 15 TIR domain
Source: IUCrJ. 2023 Apr 21;10(Pt 3):352–62. doi: 10.1107/S2052252523002956 (PMC10161769; doi:10.1107/S2052252523002956)

IUCrJ

Volume 10 (2023)

Supporting information for article:

Structural analysis of the Toll-like receptor 15 TIR domain

Kyung Yeol Ko, Wan Seok Song, Jeongho Park, Geun-Shik Lee and Sung-il Yoon

Figure S1 Structure-based sequence alignment of the chicken $TLR15_{TIR}$, human $TLR10_{TIR}$, human $TLR6_{TIR}$, human $TLR2_{TIR}$, and human $TLR1_{TIR}$ domains. The secondary structures of $TLR15_{TIR}$ are represented by arrows and waves (β-strands and α-helices, respectively) above the $TLR15_{TIR}$ sequence. The dimerization interface residues of TLR_{TIR} are colored red. The I753, Y779, and F813 residues of TLR15 that were mutated to confirm the dimerization interface are indicated by green dots.

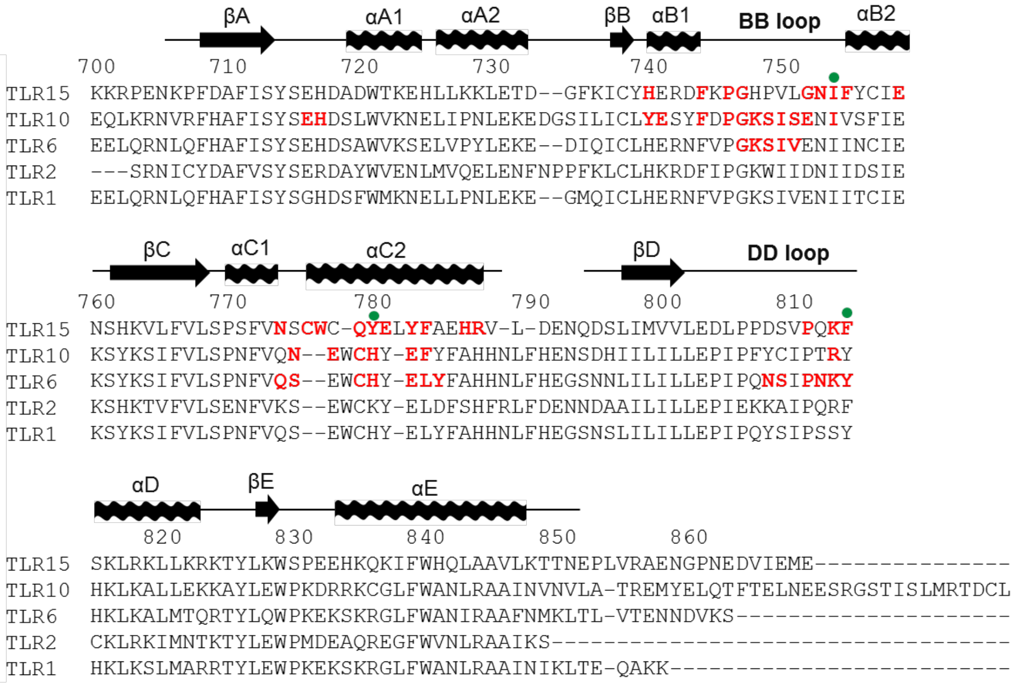

Figure S2 Overlaid TLR15$_{TIR}$ and TLR15$_{TIR\text{-}GTT}$ structures (light blue and magenta ribbons, respectively).

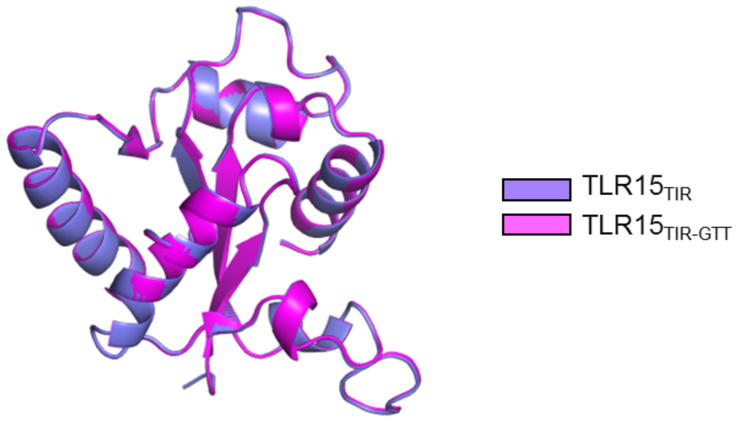

Figure S3 High sequence conservation of the dimerization interface residues and the glutathione-binding residues of chicken $TLR15_{TIR}$ (ggTLR15) in bird TLR15 (*Anser anser* TLR15, aaTLR15; *Phasianus colchicus* TLR15, pcTLR15) and reptile TLR15 (*Anolis carolinensis* TLR15, acTLR15; *Alligator mississippiensis* TLR15, amTLR15) proteins. The dimerization interface residues of chicken $TLR15_{TIR}$ are colored red, and equivalent residues in other TLR15 proteins that are identical to those in chicken TLR15 are also colored red. The glutathione-binding residues of chicken $TLR15_{TIR}$ observed in the $TLR15_{TIR\text{-}GTT}$ structure are indicated by blue dots.

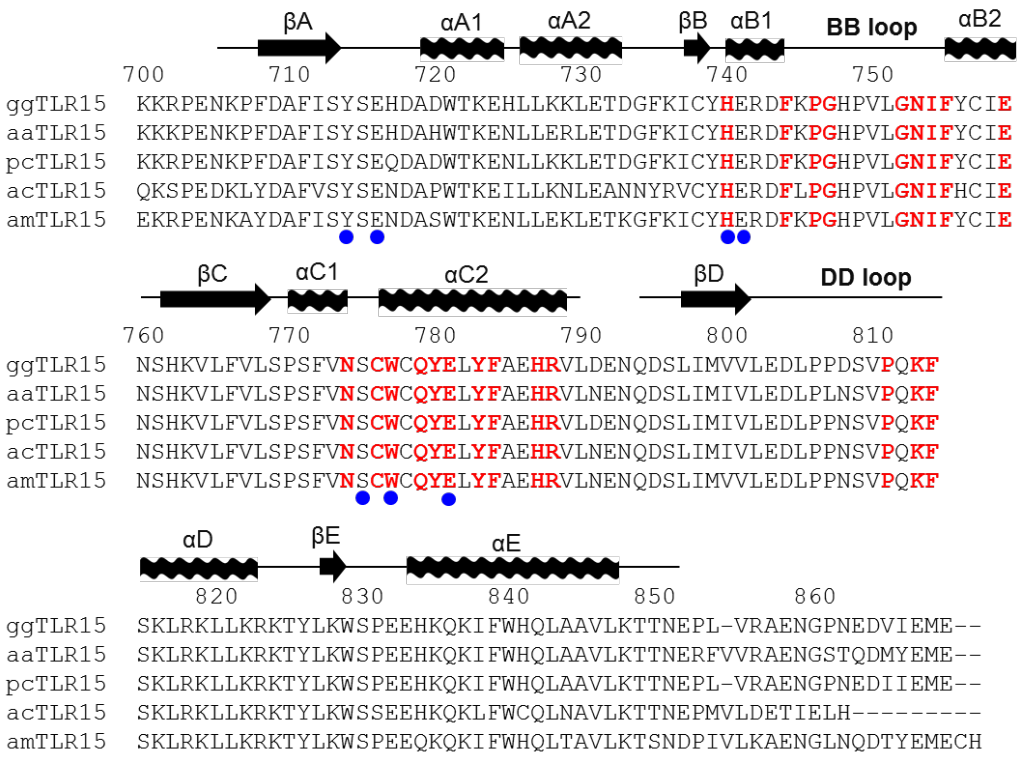

Figure S4 Detailed view of the TLR15$_{TIR}$ dimerization interface. The dimerization interface residues of TLR15$_{TIR}$ and TLR15$_{TIR}$′ are represented by green and orange sticks, respectively. The TLR15$_{TIR}$ and TLR15$_{TIR}$′ chains are shown as light blue ribbons and black Cα traces, respectively. Intersubunit hydrogen bonds are represented by black dotted lines. The orientation of this figure is identical to that of Fig. 3(*c*).

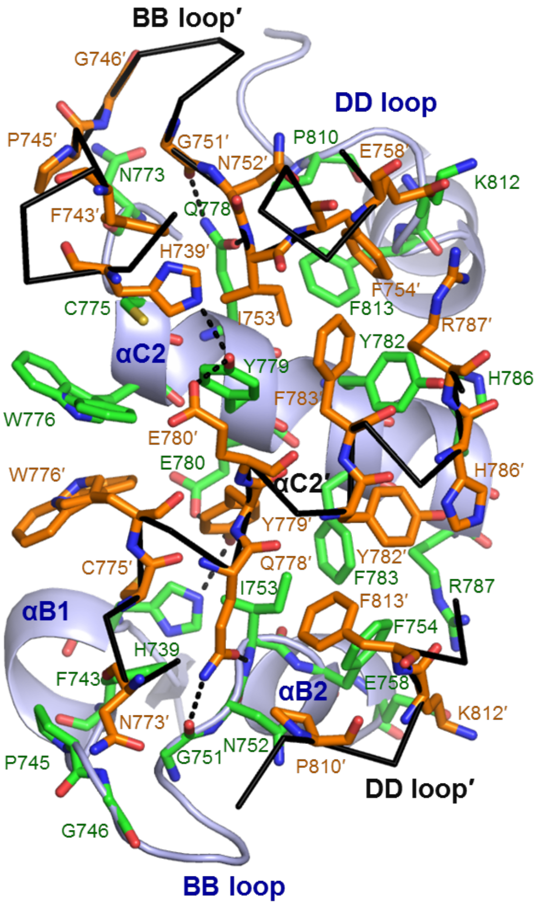

Figure S5 SDS-PAGE analysis of fractions 1-14 obtained by the gel-filtration chromatography shown in Fig. 4(*b*).

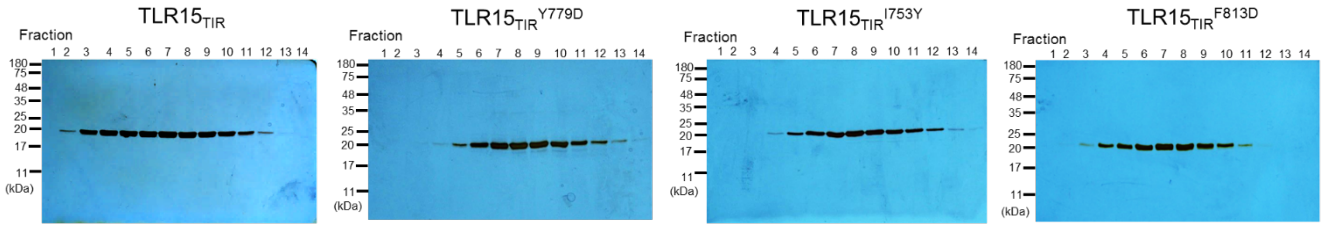

Figure S6 Overlaid structures of $TLR15_{TIR}$ (light blue ribbons), $TLR1_{TIR}$ (cyan ribbons; PDB ID 1FYV), $TLR2_{TIR}$ (yellow ribbons; PDB ID 1FYW), $TLR6_{TIR}$ (orange ribbons; PDB ID 4OM7), and $TLR10_{TIR}$ (magenta ribbons; PDB ID 2J67).

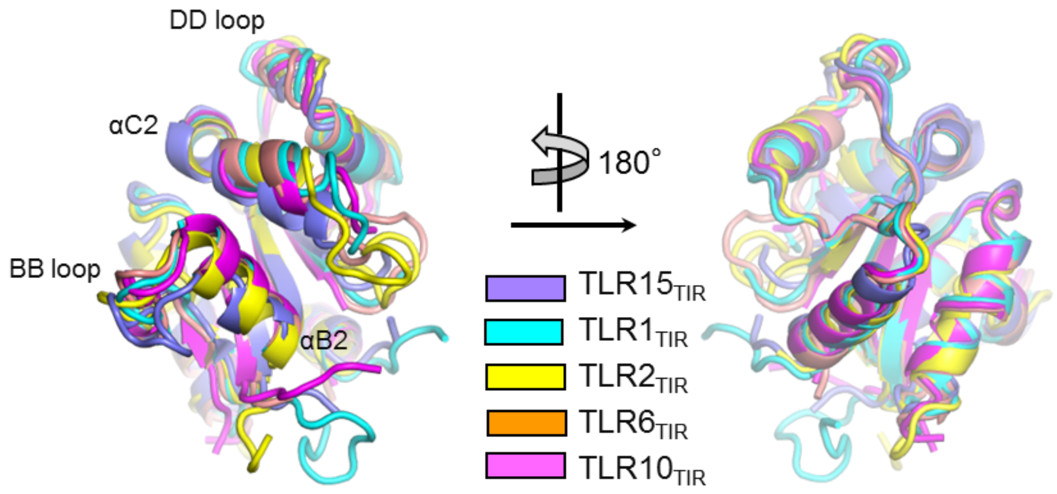

Supplement: Supplementary file 1 [file m-10-00352-sup1.pdf]
